# The Domestic Management of the Sick Room, Necessary in Aid of Medical Treatment for the Cure of Diseases

**Published:** 1841-04

**Authors:** 


Art. VII.—*The Domestic Management of the Sick Room, necessary in aid of Medical Treatment for the Cure of Diseases.* By Anthony Todd Thomson, m.d. f.l.s. &c.—*London,* 1841. 8vo, pp. 586.

We have been both pleased and disappointed with this book. We agree with the author in thinking that something of the kind was much wanted, and were therefore glad when we observed the announcement of a volume on the subject, by himself. On perusing it, however, we cannot but regret to perceive so many traces of haste, and so many departures from the proper scope of such a work. This regret is increased by the very ability with which Dr. Thomson has executed his task when treating of matters really relevant to his professed object: but when he diverges, as he often does, to matters purely professional, and fit for the consideration of medical men only, he not only encumbers his work at the risk of a positive diminution of its interest and even safety to his "domestic" readers, but from adopting a popular dress for really scientific ideas, he often falls into inaccuracies of style and expression which ought never to be indulged in. In one place, for example, he speaks of "irregular chills and heats, displaying themselves and *subsiding into a hot skin,* quick pulse, hurried or otherwise embarassed breathing." (p. 144.) Expressions of this kind are far from being unimportant; because their use raises a presumption against accuracy of thinking in any one employing them; and of all subjects on earth, medicine is that in which the greatest precision and accuracy are required. In another place, again, he says that calomel, when laid dry upon the tongue, "*hangs about the mouth and fauces,* and is more *readily taken into the habit.*" This is a very incorrect colloquialism: calomel may *adhere* to the mouth, but it can "hang about" nothing, and when absorbed it is taken into the system, but not into the "habit." But we have no wish to be severe upon Dr. Thomson, and shall therefore content ourselves with having drawn his attention to these occasional blemishes that he may correct them in subsequent editions.

As already mentioned, we have been much pleased with the simple and intelligible style in which Dr. Thomson conveys his most useful information on subjects within the legitimate scope of his work; and if he were to *excise* with a steady and unsparing hand everything by which none but professional persons can be benefited, and were then carefully to digest what is left, with a view to condensation, we feel assured that he might reduce his volume by one half, and give it a triple value to the class of readers for whom it is intended. A true "hand-book" to the sick room must be clear, concise, and to the point. If the subject is overloaded with extraneous matter the mind flags in reading it, and the essential is apt to be overlooked. If the author would keep in mind that the mother, sister, friend, or nurse is supposed to act in the sick room under the direction of the physician, and not from her own independent judgment, he would discover many pages which might be omitted with the greatest advantage. Of the 102 pages of the introduction, a very large portion is inappropriate and irrelevant; and in this respect it contrasts with the first chapter, most of which comes home to the reader as the one thing wanted. The information contained in the introduction is we admit, interesting and useful: all that we object to is, that it is out of place, and for the specific purpose of the author, is rather an encumbrance than an advantage.

In doctrine, also, we have observed one or two rather startling announcements in Dr. Thomson's book. "*Winter*," he says, "*is a more healthy season than summer*, provided we are well clothed and that we can afford fires and enjoy sufficient vigour to take exercise." (p. 6.) Is it really so? and has mankind been under a delusion for so many thousand years in looking forward to fine summer weather for the enjoyment of life and the restoration of the invalid? If our author is correct, how strongly must we have miscalculated the supposed healthful influences of the mild temperature, pure open air, sunshine, and delights of summer! But unluckily for Dr. Thomson "facts are chields that winna ding," and accordingly, on turning to the Registrar-General's last report for specific data to see whether he or we were in the wrong, we find the deaths recorded in the metropolis to stand as follows:

| | In Jan., Feb., and March, | In July, Aug., and Sept. |
|---|---|---|
| From epidemic, endemic, and contagious diseases . . . . . . . . . . | 3792 | 3141 |
| From diseases of the respiratory organs | 4715 | 2705 |
| ,, of the nervous system . . | 2255 | 1924 |
| ,, of organs of circulation . | 273 | 177 |
| ,, of digestive organs . . | 647 | 836 |
| | 11,682 | 8,783 |

Or, with the single exception of deaths from diseases of the organs of digestion which in summer exceed those in winter by 189, all the other classes of diseases are more fatal in winter than in summer in the proportion of 11,035 to 7947, or in other words, of 11 to 8; and yet, according to our author, winter is the healthiest of the two seasons, when fire, clothing, &c. are enjoyed! If he is correct, what a fearful proportion of the race must be without these advantages, even in the once "merry England."

